# Fecal Microbiota Transplantation for Fulminant Clostridioides Difficile Infection: A Combined Medical and Surgical Case Series

**DOI:** 10.7759/cureus.34998

**Published:** 2023-02-14

**Authors:** Ellen J Spartz, Mina Estafanos, Reema Mallick, Wolfganag Gaertner, Victor Vakayil, Cyrus Jahansouz, Rishav Aggarwal, Sayeed Ikramuddin, Alexander Khoruts, James V Harmon

**Affiliations:** 1 Department of Medicine, University of California Los Angeles, Los Angeles, USA; 2 Department of Surgery, University of Minnesota, Minneapolis, USA; 3 Department of Surgery, Surgery South, Stockbridge, USA; 4 Department of Medicine, University of Minnesota, Minneapolis, USA

**Keywords:** fecal microbiota transplantation in clostridium difficile infection, c-diff, c. diff colitis, fulminant c. difficile infection, total colectomy, fecal microbiota transplantation (fmt)

## Abstract

Introduction: Urgent abdominal colectomy is indicated for patients with fulminant *Clostridioides difficile* infection (CDI) when other medical therapies fail, yet mortality remains high. Fecal microbiota transplant is a less invasive alternative approach for patients with fulminant CDI. We report the 30-day complications of patients with fulminant CDI who underwent either abdominal colectomy, fecal microbiota transplantation (FMT), or FMT followed by abdominal colectomy (FMT-CO).

Methods*:* We performed a single-center, retrospective case review of combined medical and surgical patients with CDI at a large academic medical center between 2008 and 2016. Cohorts were identified as patients with fulminant CDI who underwent total abdominal colectomy alone (CO), FMT alone (FMT), or FMT-CO. We analyzed patient demographics, history, comorbidities, clinical and laboratory variables, CDI severity scores, and mortality outcomes at 30 days.

Results*:* We identified 5,150 patients with CDI at our center during the review period; 16 patients met the criteria for fulminant CDI and were included in this study, with four patients in the CO cohort, eight patients in the FMT cohort, and four patients in the FMT-CO cohort. Demographics and CDI severity scores were similar for all three groups, although the selected comorbidity profiles differed significantly among the three cohorts. The 30-day mortality rates for patients in the CO, FMT, and FMT-CO groups were 25%, 12.5%, and 25%, respectively.

*Conclusions:* FMT is an alternative or adjunctive therapy to colectomy for patients with fulminant CDI that is not associated with increased mortality. Implementation of FMT protocols in clinical practice would be dependent on the availability of qualified transplant material and successful early identification of patients likely to benefit from FMT.

## Introduction

Clostridioides difficile infection (CDI) is an ongoing worldwide public health challenge associated with substantial morbidity and mortality [1]. One of its most difficult and devastating clinical presentations is fulminant CDI. The management of fulminant CDI requires prolonged supportive care and antibiotic therapy, and frequently leads to urgent colectomy [2]. Total abdominal colectomy with end ileostomy is the most commonly performed surgical intervention (>80%) in these patients [3]. Although small series have shown that total abdominal colectomy may improve the survival of critically ill patients with fulminant CDI compared to patients receiving medical management alone, the 30-day post-colectomy mortality are as high as 50% [4].

In recent years, fecal microbiota transplantation (FMT) has emerged as a highly successful therapeutic strategy in treatment of recurrent CDI (rCDI) [5]. In the case of rCDI, normalization of the intestinal microbiome restores resistance to Clostridioides difficile by inhibiting its spore germination, expansion of vegetative cells, and production of Clostridioides difficile enterotoxins. A similar approach has been described for fulminant CDI, although the mechanisms in this context are not fully understood. Typically, repeated administrations of FMT are required to achieve a sustained cure in the treatment of fulminant CDI, in contrast to the treatment of rCDI, where a single treatment is usually sufficient to prevent recurrence. Fisher’s series included 33 fulminant CDI patients and demonstrated a cure rate of 87% with FMT without serious procedure-associated adverse events [6]. A recent systematic review and meta-analysis suggested that FMT is associated with lower mortality rates relative to what could be expected with colectomy [7]. However, the analysis was limited by heterogeneity in definitions of CDI severity, lack of patient selection and specifics of FMT protocols, and limited follow-up [7,8]. Given the chronicity of poor outcomes after abdominal colectomy in the treatment of fulminant CDI [9] and the increasing use, improved outcomes, and less treatment morbidity with FMT for CDI overall, we conducted a retrospective chart review to better appreciate patient outcomes following either FMT, total abdominal colectomy, or both in patients with fulminant CDI.

## Materials and methods

**Figure 1:**
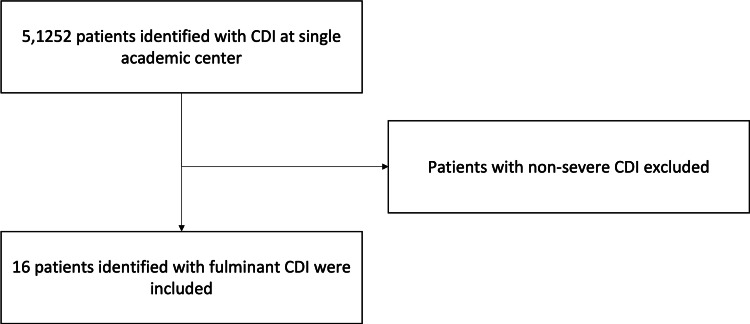
Single-center combined medical and surgical case series of patients with fulminant Clostridioides difficile infection

We describe a single-center, retrospective case series of combined medical and surgical patients with CDI at a large academic medical center from year 2008 to 2016. We identified all patients (n = 5,152) who were diagnosed with CDI during hospitalization (Figure 1). Among these patients, we identified those with fulminant CDI. Patient information was extracted from the electronic medical records, including sex, comorbidities, number of CDI episodes, and the number and type of therapeutic interventions. The ATLAS score [10], based on albumin, treatment with systemic antibiotics, white blood cell count (WBC), albumin, and serum creatinine, was calculated on all patients with fulminant CDI. The age-adjusted Charlson comorbidity index was calculated to assess disease burden due to comorbid conditions and the likelihood of mortality within one year [11]. The study protocol was approved by the Institutional Review Board.

Definitions

Severe CDI was defined by the presence of WBC > 15,000 cells/microliter or serum creatinine > 1.5 mg/dL [12]. Fulminant CDI was defined by severe CDI in conjunction with evidence of severe systemic toxicity, such as admission to the intensive care unit (ICU) for shock, fever ≥ 38.5ºC, ileus, encephalopathy, WBC ≥ 35,000 cells/microliter, elevated serum lactate levels, or associated organ failure. Patients with non-severe and severe (non-fulminant) CDI were excluded as they do not qualify for FMT per our institutional protocol. Patients with CDI and megacolon or toxic megacolon (colon dilation of at least 5 cm on abdominal imaging) were considered eligible for FMT as long as there was no evidence of bowel perforation or intra-abdominal abscess on abdominopelvic computed tomography. Successful treatment was defined as complete resolution of diarrhea, successful completion of anti-CDI therapy, avoidance of colectomy, or discharge from the hospital.

Clinical care and FMT protocol

All patients were cared for by critical care services, with consultation provided upon request by general surgeons, colorectal surgeons, gastroenterologists, and infectious disease specialists. FMT was considered after multidisciplinary discussions for the appropriateness of treatment according to the protocol [13]. FMT was typically offered when there was a consensus that total abdominal colectomy with end ileostomy constituted a prohibitive risk for the patient. Written informed consent was obtained from all patients or their legal guardians for both FMT and colectomy [14].

Our institutional FMT protocol includes several steps. First, all antibiotic therapy was discontinued for at least 24 hours, during which 2 liters of polyethylene glycol-based mechanical bowel preparation was administered via a nasogastric tube or orally. Colonoscopy was performed to confirm the presence of pseudomembranes. A thawed preparation of glycerol-cryopreserved fecal microbiota (minimum of 2.5 × 1012 bacteria) was delivered at the ascending colon. Antibiotics for additional infectious diagnoses were restarted after the procedure, and clinical progression was monitored by physical examination, a daily WBC count, and serum levels of C-reactive protein (CRP) levels. The lack of improvement within 24 hours typically promoted total abdominal colectomy with end ileostomy. If, after three days following colonoscopic administration of fecal microbiota, patients improved clinically, oral vancomycin was started, and patients were considered for discharge from the hospital. A second administration of fecal microbiota via colonoscopy was considered, at the discretion of the gastroenterologists, on an outpatient basis [15].

Outcomes and statistical analysis

The primary outcome was the 30-day mortality. FMT, age, sex, Charlson score, number of previous CDI episodes, and CDI severity were all evaluated as independent variables. Comparisons among the three groups were performed using the Wilcoxon rank sum test for continuous variables and Fisher’s exact test for categorical variables. We compared the 30-day mortality rates for patients in all three treatment groups (CO, FMT, and FMT-CO). All analyses were performed using IBM Corp. Released 2017. IBM SPSS Statistics for Windows, Version 25.0. Armonk, NY: IBM Corp.

## Results

**Figure 2:**
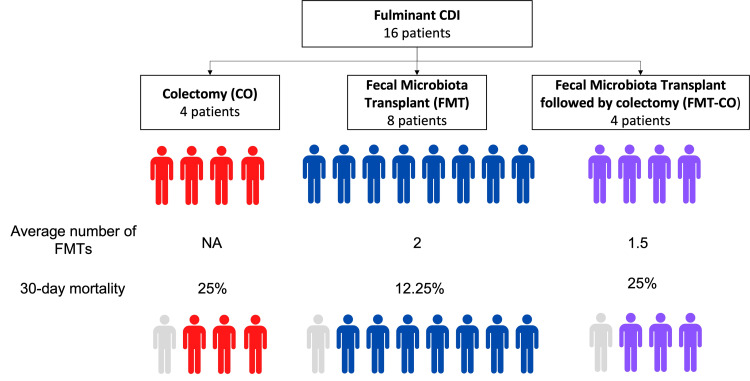
Mortality in patients with fulminant Clostridioides difficile infection based on treatment modality

A total of 5,152 patients with CDI were identified, of which 16 had fulminant CDI. Four patients underwent colectomy alone (CO), eight patients underwent FMT alone (FMT), and four patients underwent FMT followed by colectomy (FMT-CO). The median number of FMTs performed was 2 for the FMT cohort and 1.5 in the FMT-CO cohort. Patient demographics are presented in Table 1. The presence of chronic pulmonary disease and chronic immunosuppression was significantly higher in the CO and FMT-CO cohorts (p<0.05 and p<0.01, respectively). American Society of Anesthesiology (ASA) scores were significantly higher in the CO group (p <0.05). Charlson's comorbidity index was not significantly different among cohorts. Mechanical ventilation was significantly higher in the CO and FMT-CO groups compared to FMT alone (p<0.01) (Table 1). The incidence of renal failure, need for dialysis, and septic shock was not significantly different among the groups (Table 2). The overall 30-day mortality rate was 18.8%. The 30-day mortality rates for the individual groups were 25% in the CO group, 12.25% in the FMT group, and 25% in the FMT-CO group (Figure 2). The overall 60-day mortality rate for the individual groups was 50% in the CO group, 25% in the FMT group, and 25% in the FMT-CO group. 

**Table 1:** Patient demographics and baseline clinical data CO: colectomy, FMT: fecal microbiota transplant, WBC: white blood cell count, ASA: American Society of Anesthesiologists. Laboratory values reported as mean value upon initial presentation. Statistically significant values are highlighted in bold

	CO	FMT	FMT-CO	p-value
Age (mean)	67 (65, 71)	74 (60, 78)	65 (62, 72)	0.375
Male sex	25%	50%	50%	0.567
Body mass index (mean)	32 (26, 38)	30 (23, 40)	25 (22, 30)	0.363
Heart rate (beats per minute)	118 (110, 121)	94 (91, 102)	104 (96, 117)	0.185
Creatinine (mg/dL)	1.4 (0.5, 3.4)	0.64 (0.50, 1.6)	0.62 (0.47, 0.90)	0.656
WBC (x10^3^ cells/microL)	23 (1.3, 37)	21.3 (11, 36)	7.5 (3.7, 14)	0.096
Bilirubin (mg/dL)	0.9 (0.2, 1.7)	0.4 (0.2, 1.8)	0.4 (0.3, 0.5)	0.797
AST (U/L)	46.5 (21, 100)	26 (13, 59)	16 (14, 27)	0.208
ALT (U/L)	37 (10, 52)	21 (12, 24)	18 (13, 27)	0.196
Pulmonary disease	100%	50%	0%	< 0.05
Cardiac history	75%	50%	50%	0.695
Endocrine disorder	50%	75%	75%	1.0
Current antibiotic use	100%	75%	100%	1.00
Immunosuppression	75%	0%	75%	< 0.01
Charlson Comorbidity Index	4.5 (3, 5)	4 (3 ,5)	5.5 (3.5, 7.5)	0.462
ASA Score	5 (4, 5)	3 (3, 4)	3 (3, 4)	< 0.05
ATLAS Score	6 (4, 7)	5 (4, 7)	6 (4, 6)	0.929

**Table 2:** Patient co-morbidities at the time of treatment CO: colectomy, FMT: fecal microbiota transplant. Statistically significant values are highlighted in bold.

	CO	FMT	FMT-CO	p-value
Respiratory failure requiring mechanical ventilation	100%	25%	75%	< 0.01
Acute renal failure	25%	50%	50%	0.850
Septic shock	100%	75%	75%	0.368
Hemodialysis	0%	12.5%	0	1.00

## Discussion

Although colectomy largely remains the standard of care in the treatment of fulminant CDI, recent literature suggests that FMT can be an effective nonoperative option. FMT for CDI was introduced at our institution in 2011, which provided an opportunity to examine its impact on the outcomes of patients with fulminant CDI. All FMT patients were initially evaluated with a multidisciplinary approach, and a joint decision was made to attempt FMT, given the high operative risk profile and poor overall health status in this selective subset of patients. One-fourth of the entire group underwent colectomy alone. One-third of patients who received FMT ultimately underwent colectomy due to inadequate response to FMT. Patients who underwent immediate colectomy alone were not considered for subsequent FMT. Although difficult to fully know given the design of our study, the potential reasons for immediate colectomy include a lack of awareness of the FMT option at our institution and precipitous clinical deterioration that did not allow sufficient time to consider FMT. Ultimately, the 30-day mortality rates were comparable among the three patient groups in our case series.

FMT appears to be an effective therapy for select patients with severe or fulminant CDI, although the current literature has shown mixed results with regard to mortality. Given that fulminant CDI encompasses only 3%-5% of all patients with CDI [16], large studies are often challenging to perform. A recent systematic review and metanalysis on FMT for severe and fulminant CDI, including 16 studies, showed an overall clinical cure rate for FMT of 61%, an 11% rate of major adverse events, an 8% rate of colectomy, and a pooled all-cause mortality rate after FMT of 16% [7]. This mortality rate is similar to our findings, with a mortality of 12% for patients who received FMT alone and 25% for patients who underwent FMT followed by colectomy.

The current literature also supports the effectiveness of FMT as a treatment option for recurrent or refractory CDI, with cure rates ranging from 70% to 90% [4]. Furthermore, a cost-effective analysis indicated that FMT demonstrated lower cost and higher quality-adjusted life years compared to standard therapy, even when multiple FMTs were required [17]. Currently, this option may be limited by the availability of qualified FMT materials. FMT material is always available for emergent procedures at our center; however, we are continuing to increase awareness of the utility of FMT for patients with fulminant CDI. This also requires system-wide protocols in order to be successful. 

This study has many noticeable limitations, including its retrospective nature, limited numbers, and selection bias based on disease presentation, treatment availability, patient co-morbidities, and clinical status, as well as managing treatment disease. Notably, many patients included in this case series were critically ill. Also, at our institution, gastroenterology or surgical consultations typically result in earlier consideration of FMT. At our institution, inpatient FMT is limited to patients with fulminant CDI. However, there may be a benefit for early FMT for hospitalized patients with severe CDI rather than only patients with fulminant CDI, with studies showing improved survival at short-term (three-month) follow-up [10,18]. One retrospective study included patients with both severe and fulminant CDI who required treatment in an intensive care unit and showed a mortality benefit for patients who received FMT with a number needed to treat three patients to prevent a single death [19]. Another retrospective series examined mortality in patients with either fulminant CDI or severe refractory CDI before and after implementation of an inpatient FMT program; the authors found that CDI-related mortality was significantly decreased in both cohorts of patients after implantation of the inpatient FMT program [4]. A retrospective series reported mortality in patients with severe or fulminant CDI who received FMT and demonstrated reduced mortality when evaluated using univariable analysis but not when evaluated by multivariable analysis; these findings may have been due to the small sample size [20].

**Figure 3:**
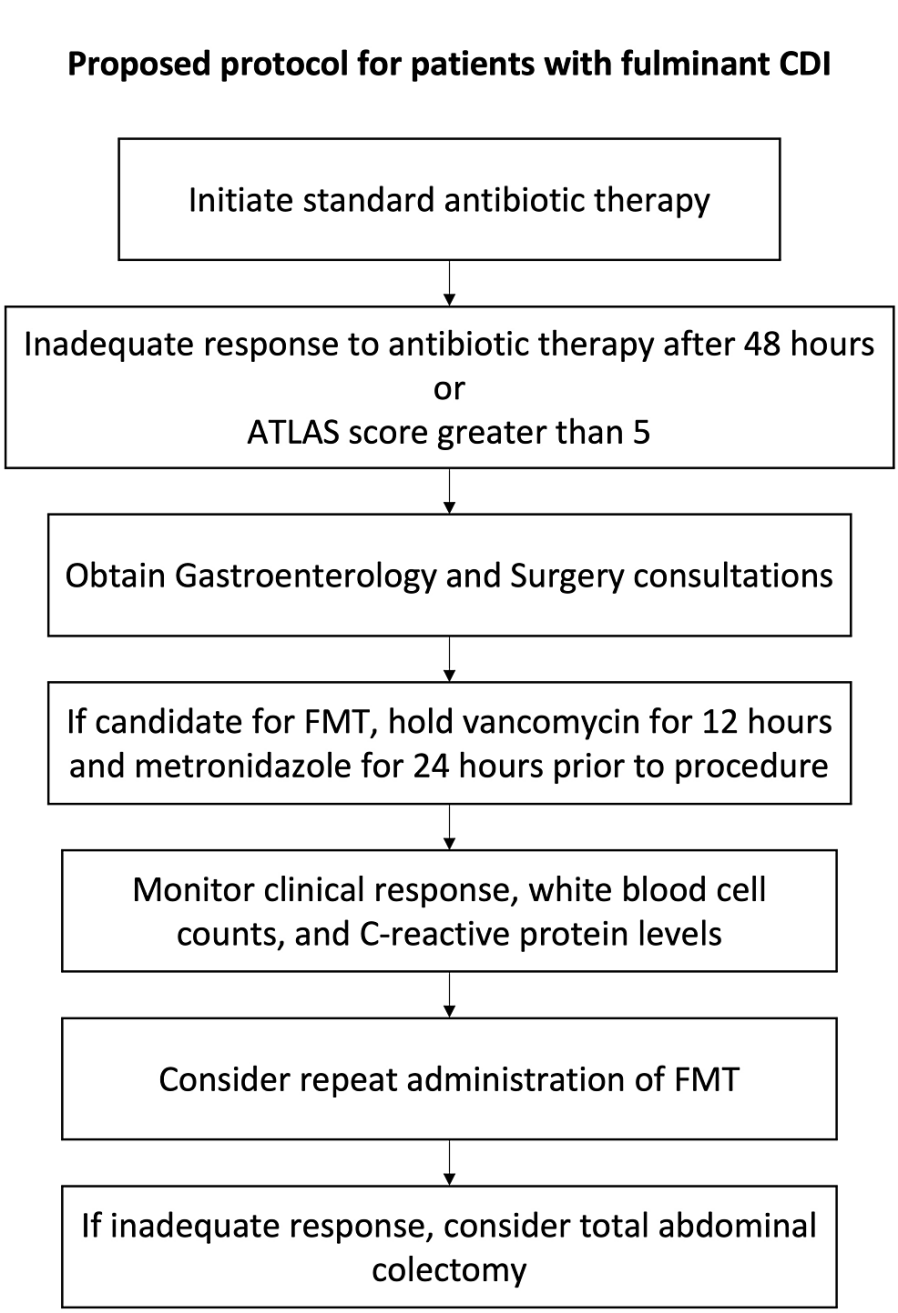
Our proposed protocol and patient care algorithm for patients with fulminant Clostridioides difficile infection

The optimal protocol for FMT in fulminant CDI has not yet been defined. At our medical center, we discontinue all antibiotics before the procedure, and the patient response is measured largely through the patient's clinical status, including frequency and volume of diarrhea, hemodynamic stability, abdominal examination, abdominal imaging, and laboratory data, instead of the disappearance of pseudomembranes on repeat colonoscopies (Figure 3). Repeated administration of FMT is common, given the reported high rates of CDI recurrence with just a single administration of FMT [21]. Further investigation is needed to identify factors associated with FMT treatment failure. The surgical approach at our institution involves total abdominal colectomy with end ileostomy. Although there remains significant interest in the literature regarding the option of performing a loop ileostomy to perform antegrade vancomycin administration via the efferent stoma limb [22], we have not pursued this approach in our center. A retrospective study using a national database examined outcomes for patients with fulminant CDI who underwent partial colectomy versus total abdominal colectomy; significant differences in mortality, postoperative complications, or length of hospital stay were not identified; however, overall 30-day mortality remained high in both groups [23]. Current guidelines by the Surgical Infection Society recommend total abdominal colectomy as the procedure of choice for severe or fulminant non-perforated CDI, as diverting loop ileostomy with antegrade colonic vancomycin has been associated with higher rates of recurrent CDI after ostomy reversal [24]. 

Despite the limitations of our study, we demonstrated that FMT is not associated with worse mortality in select patients with fulminant CDI and therefore adds to the existing literature on the role of FMT in treating fulminant CDI. Future research, including prospective studies, is warranted to better understand patient identification and selection, as well as the true impact of FMT in patients with fulminant CDI. Furthermore, our report supports the inclusion of FMT into management protocols for fulminant CDI, which could spare patients from the morbidity associated with colectomy and possibly lead to improved survival. 

## Conclusions

This single institution, a retrospective study, showed that FMT can be a safe alternative or adjunct to colectomy in select patients with fulminant CDI. Early implementation of multidisciplinary FMT protocols is crucial for the selection and management of this challenging group of patients. FMT alone was associated with a lower mortality rate in patients with fulminant CDI. FMT may become an alternative approach for patients with fulminant CDI before considering surgical intervention. 
